# A longitudinal study of the faecal microbiome and metabolome of periparturient mares

**DOI:** 10.7717/peerj.6687

**Published:** 2019-04-03

**Authors:** Shebl E. Salem, Rachael Hough, Chris Probert, Thomas W. Maddox, Philipp Antczak, Julian M. Ketley, Nicola J. Williams, Sarah J. Stoneham, Debra C. Archer

**Affiliations:** 1 Department of Epidemiology and Population Health, Institute of Infection and Global Health, University of Liverpool, Leahurst campus, Wirral, UK; 2 Department of Surgery, Faculty of Veterinary Medicine, Zagazig University, Zagazig, Al Sharquiya, Egypt; 3 Department of Cellular and Molecular Physiology, Institute of Translational Medicine, University of Liverpool, Liverpool, UK; 4 Department of Musculoskeletal Biology, Institute of Ageing and Chronic Disease, University of Liverpool, Liverpool, UK; 5 Computational Biology Facility, Institute of Integrative Biology, University of Liverpool, Liverpool, UK; 6 Department of Genetics and Genome Biology, College of Life Sciences, University of Leicester, Leicester, UK; 7 TopSpec Equine Ltd, Middle Park Farm, North Yorkshire, UK

**Keywords:** Horse, Mare, Colic, Microbiota, Microbiome, Metabolome, VOCs

## Abstract

**Background:**

Periparturient mares are at increased risk of colic including large colon volvulus, which has a high mortality rate. Alterations in colonic microbiota related to either physiological or management changes, or both, that occur at this time have been suggested as potential causes for increased colic risk in this population of horses. Although the effect of management changes on the horse faecal microbiota has been investigated, limited work has been conducted to investigate changes in faecal microbiota structure and function in the periparturient period. The objectives of the current study were to investigate temporal stability of the faecal microbiota and volatile organic compounds (VOCs) of the faecal metabolome in periparturient mares.

**Methods:**

Faecal samples were collected weekly from five pregnant mares from 3 weeks pre-foaling to 7 weeks post-foaling. The microbiome data was generated by PCR amplification and sequencing of the V1–V2 regions of the bacterial 16S rRNA genes, while the VOC profile was characterised using headspace solid phase microextraction gas chromatography mass spectrometry.

**Results:**

The mare faecal microbiota was relatively stable over the periparturient period and most variation was associated with individual mares. A small number of operational taxonomic units were found to be significantly differentially abundant between samples collected before and after foaling. A total of 98 VOCs were identified. The total number of VOCs did not vary significantly between individual mares, weeks of sample collection and feeds available to the mares. Three VOCs (decane, 2-pentylfuran, and oct-2-ene) showed significant increase overtime on linear mixed effects modelling analysis. These results suggest that the mare faecal microbiota is structurally and functionally stable during the periparturient period. The findings also suggest that if changes in the gut microbiota are related to development of colic postpartum, altered risk may be due to inherent differences between individual mares. VOCs offer a cost-effective means of looking at the functional changes in the microbiome and warrant further investigation in mares at risk of colic.

## Introduction

Parturition and the periparturient period have been shown to be associated with increased risk of colic in several studies (Kaneene et al., 1997). Postpartum colic occurred in 11% of broodmares in a longitudinal study (Weese et al., 2015) and 36% of these cases were due to large colon volvulus (LCV). LCV is a form of colic that is associated with high mortality (Driscoll et al., 2008; Harrison, 1988) and increased risk of post-operative colic (French et al., 2002). Broodmares were 13 times more likely to develop LCV in one case control study (Suthers et al., 2013) and represented 74–77% of horses undergoing surgical management of LCV in two case series (Ellis et al., 2008; Snyder et al., 1989). Furthermore, around one-fifth of colic surgeries performed over a 24-year-period in a US equine referral hospital were for the correction of LCV in Thoroughbred mares (Hackett et al., 2014). Broodmares appear to be at greatest risk during the early postpartum period based on the fact that approximately 30% of horses diagnosed with LCV in two studies were postparturient mares that had foaled within 90 days prior to hospital admission (Snyder et al., 1989; Suthers et al., 2013).

The periparturient period is characterised by frequent changes in management including feeding practices, which have been consistently identified as risk factors for colic in general (Cohen, Gibbs & Woods, 1999; Cohen et al., 1995; Cohen & Peloso, 1996; Hudson et al., 2001) and for LCV (Suthers et al., 2013). It has been hypothesised that dietary and other management changes may disrupt colonic microbes and may induce changes in colonic pH and volatile fatty acid production, predisposing horses to intestinal dysfunction (Cohen, Gibbs & Woods, 1999; Hudson et al., 2001). Although perturbation of the horse gut microbial communities (known as gut microbiota) as a result of diet change is well-documented in microbiological studies (Daly et al., 2012; Fernandes et al., 2014), the effect of foaling or any associated hormonal or metabolic changes that occur during the periparturient period in these microbial communities is largely unknown.

Relatively little research has been conducted to determine if there are changes in the equine gut microbiota associated with foaling. Given the association between recent foaling and colic, and LCV in particular, this would provide valuable information about the potential role of gut microbiota in colic development. Faecal samples collected from broodmares at four time points (14 days prior to foaling and 4, 14, and 28 days post-foaling) in one study revealed stability of the faecal microbiota in this group of mares compared with non-pregnant control mares and when pre- and postpartum samples were compared (Weese et al., 2015). However, the risk period for postpartum colic in broodmares appears to be greatest in the first 90 days post-foaling and the latter study did not investigate this longer period.

Volatile organic compounds (VOCs) are the products of metabolism of the microbiota and the host (mare). These compounds provide information about the functional microbiota (i.e. what the bacteria produce), which may be more important than identification of bacteria alone. In humans and animals, altered VOC profiles were shown to be non-invasive indicators of gastrointestinal disease (Garner et al., 2007; Leng et al., 2018). Therefore, faecal VOC analysis may eventually provide a monitoring tool for types of equine colic that may arise from gut dysbiosis. The aim of the current study was to characterise the faecal microbiota and the faecal volatile metabolome of periparturient mares from 3 weeks pre-foaling to 7 weeks post-foaling, and to explore temporal stability of structure of the microbial community and the VOCs it produces.

## Materials and Methods

### Mares

Five healthy pregnant mares from the same farm were recruited onto the study approximately 1 month prior to their foaling due dates. The mares had no history of colic or other medical conditions during pregnancy. The demographics of the mares are summarised in Table S1. The mares’ diet was supplemented with a feed balancer (Opti-Care Balancer; Gain Equine Nutrition, Durham, UK) throughout the study and they had free access to water from automatic watering devices. Pre-foaling and during the first few weeks post-foaling the mares were managed on grass paddocks during the day and stabled at night in separate foaling boxes where they were fed hay. Mares were then turned out in groups in larger grass paddocks with free access to lush grass until the end of the study. The timing of introduction of each of these nutritional management practices varied between mares and details are given in Fig. S1. Moxidectin (Equest, Zoetis, Surrey, UK) was administered to all mares approximately 2 months prior to collection of the first set of samples, which was part of the normal management routine of the mares. The study was approved by The University of Liverpool Veterinary Research Ethics Committee (VREC207) and the manager of the farm consented to participate.

### Sample collection

A total of 11 samples (approximately 200 g each) were collected weekly from faeces passed by individual mares immediately following observed defaecation (samples labelled T-3 to T7). Samples were placed in plastic sealable bags and were stored in a refrigerator (4 °C) at the farm temporarily until all samples had been collected from mares scheduled for sampling on that day. Samples were then transferred to a −80 °C freezer where they were stored until processing. All samples were frozen within 5 h of collection except samples collected during the second stage labour (T0) where the yard staff collected these samples and refrigerated them until the next visit by the principal investigator. The sampling schedule and types of feed at each sampling occasion are depicted in Fig. S1.

### DNA extraction and generation of sequence data

A commercial kit (QIAamp DNA Stool Mini Kit; QIAGEN, Manchester, UK) was used to extract DNA from samples followed by PCR amplification of the V1–V2 hypervariable regions of the bacterial 16S ribosomal ribonucleic acid (rRNA) gene to create amplicon libraries for sequencing using the Ion Torrent Personal Genome Machine system. Details of sample preparation and generation of sequence data are described previously (Salem et al., 2018).

### Faecal volatile organic compound profiling

The VOC profile of faeces was determined using headspace solid phase microextraction gas chromatography mass spectrometry (HS-SPME-GCMS) using the methods described by Hough, Archer & Probert (2018).

### Data analysis

#### Microbiome data analysis

The data generated from sequencing of the 16S rRNA gene amplicon libraries were processed using the QIIME pipeline (version 1.8.0; http://qiime.org/) (Caporaso et al., 2010b). Sequences from different samples were demultiplexed according to their barcode sequences and chimeric sequences were identified using the UCHIME algorithm (Edgar et al., 2011) and were filtered from the data. Sequences were then clustered open-reference into operational taxonomic units (OTUs) at 97% identity threshold using USEARCH (version 6.1.544) (Edgar, 2010). A representative sequence for each OTU cluster was aligned to the Greengenes core set (version 13.8) (DeSantis et al., 2006) using PyNAST (Caporaso et al., 2010a), filtered to remove gaps and hypervariable regions using the Lane mask before creating an approximately-maximum-likelihood phylogenetic tree using FastTree (Price, Dehal & Arkin, 2010). Taxonomic assignment of OTU representatives was performed using the ribosomal database project classifier (version 2.2) (Wang et al., 2007) informed with the Greengenes reference database at a 50% confidence limit.

Statistical analyses were performed using R software environment (version 3.2.2) (R Core Team, 2014) with the following add-on statistical packages: ‘phyloseq’ (McMurdie & Holmes, 2013), ‘vegan’ (Oksanen et al., 2015), ‘ggplot2’ (Wickham, 2009), ‘nlme’ (Pinheiro et al., 2015), and ‘cluster’ (Maechler et al., 2016). Apart from alpha diversity analysis, the OTU table was further filtered to remove low-abundance OTUs (OTUs present in <5% of samples or represented by <20 reads) and normalised by rarefying to account for unequal sequencing effort between samples (Weiss et al., 2015).

The data were clustered hierarchically based on the average linkage agglomerative clustering method (UPGMA) following calculation of a Bray–Curtis dissimilarity matrix. The trees were visualised using publicly available software (FigTree version 1.4.2). Principal coordinate analysis (PCoA) was performed on a Bray–Curtis dissimilarity matrix created from the OTU table. The amount of variation in the data that could be explained by either the time of sample collection relative to foaling, feed or the individual mares was estimated using permutational multivariate analysis of variance (PERMANOVA) following calculation of a Bray–Curtis dissimilarity matrix from the data using the ‘vegan::adonis’ function in R.

The statistical methods and R script used for investigating changes in faecal microbiota diversity and stability over time in the current study were adapted from publicly available R codes (DiGiulio et al., 2015a). Alpha diversity analysis involved calculation of Chao1 index (Chao, 1984) for species richness (number of different species within a community); and Shannon (Shannon, 1948) diversity index for population diversity (a measure of species richness and similarity of species abundance within a community). The pattern of change of calculated measures over time relative to foaling were evaluated using linear mixed-effects modelling (LME). Random intercept and slope LME models were fitted where mares were included as a random effect and time relative to foaling (in weeks) was included as a fixed effects term in the model.

Beta diversity analysis involved calculation of weighted-UniFrac (Lozupone et al., 2007) and Bray–Curtis (Bray & Curtis, 1957) dissimilarity metrics. Distances between consecutive samples of the community (distances between consecutive sampling time points) within each of the mares were calculated and were used as a measure of stability of the community over time relative to foaling. The calculated distances were modelled using random intercept and slope LME models where mares were treated as a random effect variable and where time was the fixed portion of the model.

Samples collected during a 3-week period before and after foaling, were compared for differentially abundant OTUs using negative binomial models. The models were fitted using the DESeq2::DESeq function in R. Prior to differential abundance analysis, the OTU table was further filtered to exclude OTUs present in <25% of the samples. *p-*values were adjusted for multiple testing using the false discovery rate (FDR) method (Benjamini & Hochberg, 1995). OTUs the adjusted *p*values of which were <0.1 were considered significant. Results from this model were presented in a dot plot.

#### Metabolome data analysis

Metabolome data were processed using Automated Mass Spectral Deconvolution System (AMDIS-version 2.71, 2012) and VOCs were putatively identified using the National Institute of Standards and Technology mass spectral library (version 2.0, 2011). Data were aligned using the R package Metab (Aggio, Villas-Boas & Ruggiero, 2011). All samples were analysed in triplicate, an average was taken of the technical replicates and taken forward for data analysis. To allow statistical comparison of relative compound abundance, any missing values present after taking an average were replaced with a half-minimum value of the data matrix. Clustering within the data was investigated using principal component analysis (PCA) and the mean number of VOCs identified were compared between individual mares, weeks of sample collection and the types of feeds using ANOVA followed by Tukey’s HSD test. PERMANOVA was used to estimate the amount of variation in the data that could be explained by individual mares, week of sample collection and the type of feed. LME modelling was used to explore the pattern of change of individual VOCs over time. For each VOC a Random intercept model was fitted where mares were included as a random effect variable and time in weeks was included as a fixed effects term in the model. VOCs with *p*-values of <0.1 following adjustment for multiple comparisons using the FDR method were considered significant. All analyses were performed using R (version 3.2.2).

## Results

### Mare faecal microbiota

**Figure 1:**
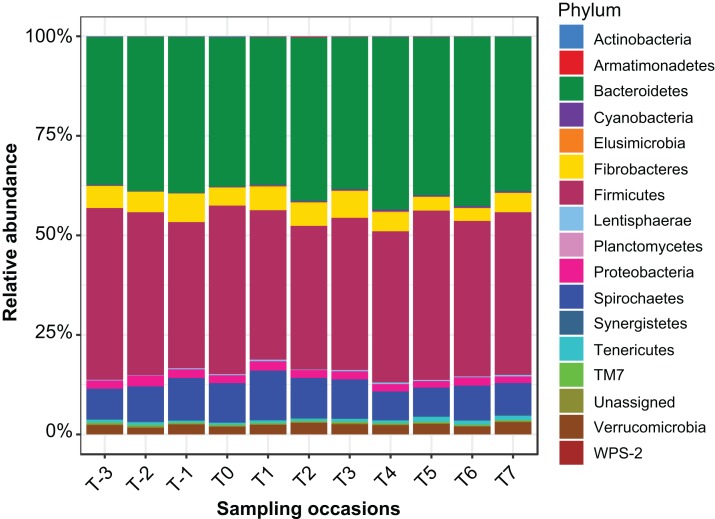
Relative abundance of bacterial phyla identified in the mare faecal microbiota. A bar plot of the relative abundance of different bacterial phyla identified in the data.

Sequencing of PCR-amplified 16S rRNA genes from 55 samples resulted in 1,648,876 quality non-chimeric sequences. Each sample had at least 17,392 reads, and there were an average of 29,980 reads per sample. The reads were clustered into 17,863 OTUs. Filtration of spurious OTUs reduced this count to 7,843 OTUs (40% of the original count). In this filtered OTU count table, 16 bacterial phyla were identified (Fig.1), the relative abundance of which at each sampling time point is presented in Table S2.

**Figure 2:**
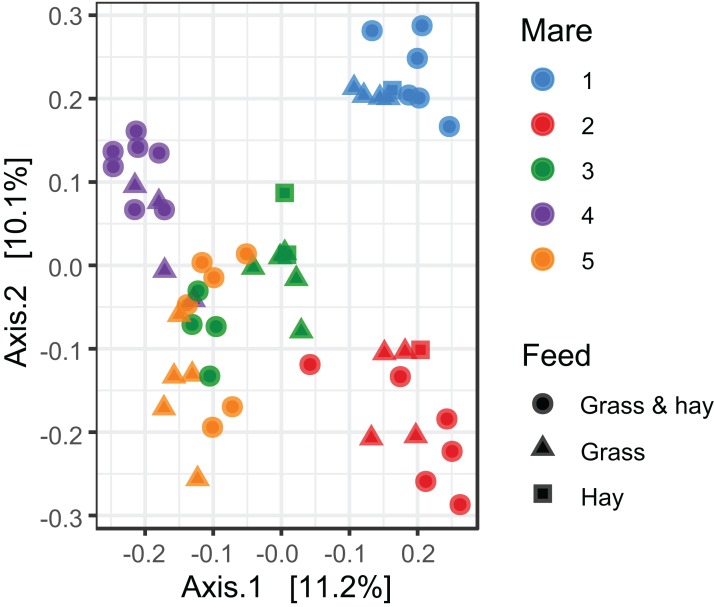
Principal coordinate analysis of the mare faecal microbiota. Ordination plot of the first two axes from the principal coordinate analysis (PCoA) of microbiome data. Data points are coloured by individual mares and shaped by the types of feed.

Cluster analysis revealed that the data were clustered by mares rather than by time relative to foaling. UPGMA trees built from a Bray–Curtis dissimilarity matrix is provided in Fig. S2. A similar pattern of clustering was also confirmed in a PCoA plot (Fig. 2). PERMANOVA analysis revealed that 3% (*R*^2^ = 0.03, *p-*value = 0.001) of variation in the data could be explained by the variable time, 5% (*R*^2^ = 0.05, *p-*value = 0.001) by the type of feed and 33% (*R*^2^ = 0.33, *p-*value = 0.001) by individual mares.

**Figure 3:**
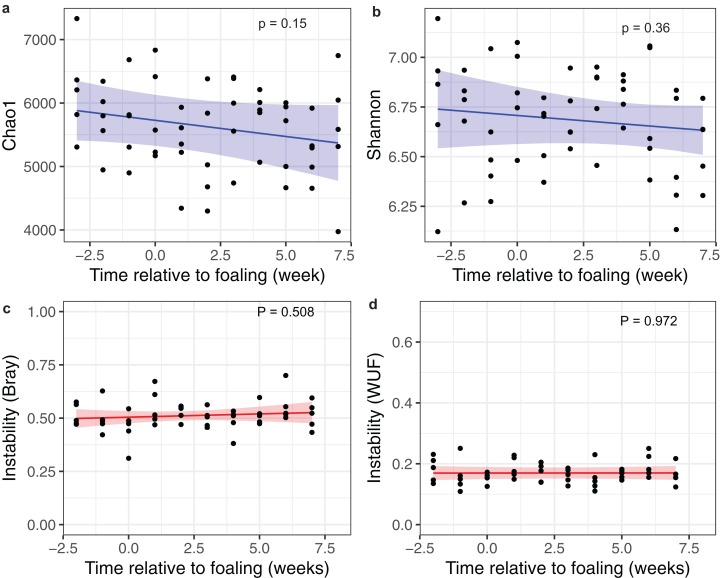
Changes in alpha and beta diversities over time. Prediction plots of (A) Chao1 and (B) Shannon diversity measures; and (C) a Bray–Curtis and (D) a Weighted Unifrac dissimilarity matrices against time relative to foaling. Blue and red lines are the regression lines from the linear mixed-effects models and cognate shading denotes the 95% confidence limits of the prediction. The models included time as a fixed effects variable and the mares as a random effect variable.

Linear mixed-effects modelling of Chao1 and Shannon index diversity measures revealed general stability of alpha diversity over time. Prediction plots from these models are given in Figs. 3A and 3B. Similar results were also obtained when investigating dissimilarity (beta diversity) between consecutive sampling time points (Figs. 3C and 3D). These results suggest that the mare faecal microbiota was stable from 3 weeks pre-foaling to 7 weeks post-foaling. Only 81 OTUs were found to be significantly differentially abundant between samples collected during a 3-week period prior to foaling and a 3-week period post foaling (Fig. S3). These results also confirm greater stability of the mare faecal microbiota around the time of foaling. Of these OTUs, 54 were upregulated and 27 were downregulated post-foaling. Downregulated OTUs belonged mainly to the Ruminococcaceae family (*n* = 39) while the upregulated OTUs were represented mainly by members of Spirochaetaceae family (*n* = 6) (Table S3).

### Mare faecal metabolome

**Figure 4:**
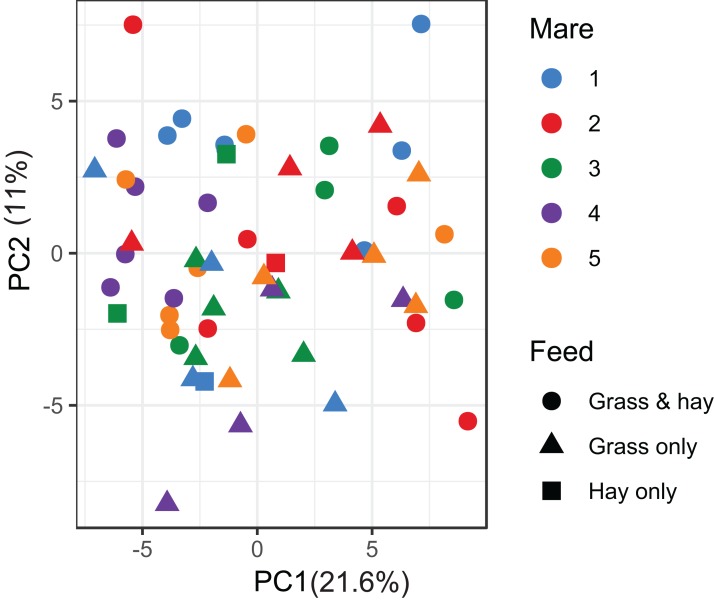
Principal component analysis of metabolome data. Ordination plot of the first two axes from the principal component analysis (PCA) of metabolome data. The plot shows that there is no clear clustering of the data by either the individual mares or the types feeds available to the mares. Data points are coloured by individual mares and shaped by the types of feeds.

**Figure 5:**
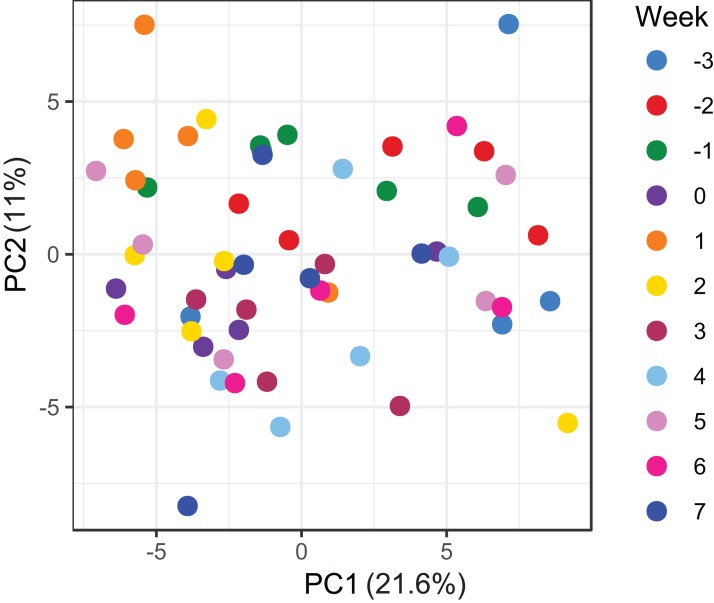
Principal component analysis of metabolome data. Ordination plot of the first two axes from the principal component analysis (PCA) of metabolome data. The plot shows that there is no clear clustering of the data by sampling time points. Data points are coloured by the time of sampling relative to foaling.

**Figure 6:**
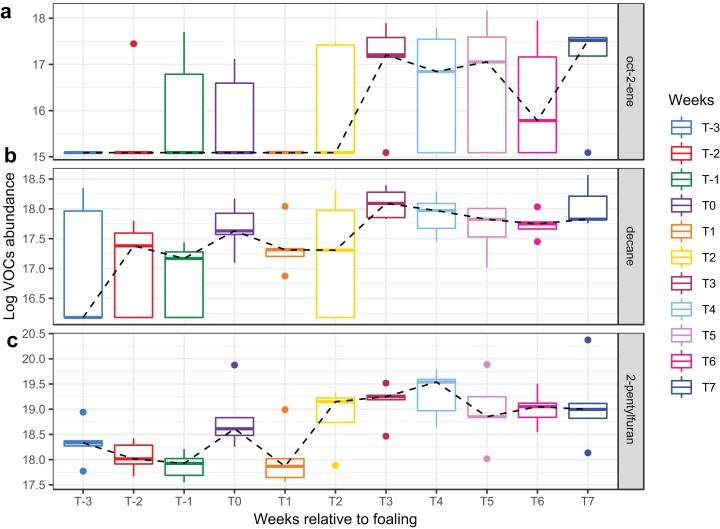
Boxplots of three VOCs that significantly increased over time during the study period. Boxplots of three VOCs ((A) oct-2-ene, (B) decane, (C) 2-pentylfuran) that significantly increased over time during the study period. Changes of VOC abundance over time was modelled using linear mixed-effects modelling with the mare included as a random effect variable and the time (in weeks) as a fixed effects variable in the model.

A total of 98 VOCs were characterised in the faecal metabolome in all samples. The mean number of VOCs did not differ significantly between individual mares, weeks of sample collection or the types of feed (Figs. S4A–S4C). There was no clear clustering of the data on PCA analysis (Figs. 4 and 5). Similar to the microbiome data, PERMANOVA analysis showed that a small amount of variation could be explained by the time of sampling (*R*^2^ = 0.05, *p* = 0.01) and type of feed (*R*^2^ = 0.06, *p* = 0.04), while individual mares accounted for 12% of variation in the data (*R*^2^ = 0.12, *p* = 0.04). LME modelling identified three VOCs which increased significantly (adjusted *p-*value < 0.1) over time during the study period (Fig. 6). These were decane, 2-pentylfuran and oct-2-ene.

## Discussion

The current study has characterised the faecal microbiota and the faecal metabolome of a group of pregnant mares during the periparturient period, at times when broodmares are at greatest risk of colic. We found that these microbial communities were structurally and functionally stable over the course of the study, and changes that were identified were largely associated with individual mares rather than being related to the time of sample collection. These findings are in agreement with previous studies (Blackmore et al., 2013; Proudman et al., 2015) and suggest that if changes in faecal microbiota are associated with postpartum colic, altered risk may be due to inherent differences between individual mares.

The findings of the current study are consistent with a recent human study that investigated the microbiota composition of four different body sites including the distal gut, vagina, saliva, and tooth/gums of pregnant women (DiGiulio et al., 2015b). The latter study found that these microbial communities were stable throughout pregnancy and post-delivery. Our findings are also consistent with those reported by Weese et al. (2015) who reported a minimal effect of foaling on the mare faecal microbiota. None of the mares included in the current study developed colic either pre- or postpartum and therefore the results reported here cannot be related directly to colic occurrence. However, our results suggest that it is unlikely that inherent changes in faecal microbiota of mares during the periparturient period are a major contributing factor in the development of periparturient colic. Weese et al. (2015) reported significant increase in relative abundance of the phylum Proteobacteria and reduction in relative abundance of the Firmicutes, Bacteroidetes, and Tenericutes phyla prior to the onset of postpartum colic. This observation requires further investigation to determine if such markers can identify mares at increased risk of colic and any interventions that may reduce this risk.

The level of alpha diversity of the mare faecal microbiota exhibited an overall linear stability with time relative to foaling. Alpha diversity is a measure of within-sample biodiversity and is often used to associate the perturbation of microbial communities with a disease process or changes in the physiological status of the host (De Weerth et al., 2013; Elli, Colombo & Tagliabue, 2010; Turnbaugh et al., 2009). Changes in beta diversity overtime were also minimal which together suggest that the mare faecal microbiota exhibited minimal change during the period under investigation. The significant increase in mean count of OTUs that belonged to the Ruminococcaceae family and the decrease in members of Spirochaetaceae family post-foaling could be attributed to changes in feed types where mares were predominantly fed on grass post-foaling. These findings concur with a previous study, which reported increase in relative abundance of members of Ruminococcaeae and decrease in relative abundance of members of Spirochaetaceae bacterial families in response to grass feeding (Salem et al., 2018).

Three compounds (decane, 2-pentylfuran, and oct-2-ene) altered significantly with time in the current study, while the remainder of the faecal VOC metabolome of the mares remained stable.

Decane, 2-pentylfuran, and oct-2-ene have been detected from the faecal headspace of ruminants and they may originate directly from food or as products of microbial fermentation (Cai et al., 2006; Fischer et al., 2015; Garner et al., 2007; Schulz & Dickschat, 2007). Therefore, the significant changes in these VOCs in the current study may reflect a change in feed type or mare feed intake over time. In general, the faecal VOC metabolome appeared to be more uniform among individual mares than the faecal microbiota, indicating a common functionality between bacteria. Overall, the microbiome and metabolome mirrored each other, in that they both remained stable, indicating that VOCs may be a cost-effective alternative to monitoring the faecal microbiome.

In the current study, the faecal microbiota was used as a proxy for the hindgut microbial populations. Studies that compared the microbiota composition of different regions of the horse gastrointestinal tract have found that faecal microbiota partially represent the microbial populations of the large intestine, particularly those of the distal colon (Costa et al., 2015; Dougal et al., 2012). Given that it is not possible to directly sample the colonic microbiota sequentially, faecal analysis is the best possible proxy measure of potential changes in the colonic microbial community. The effect of a new diet on the equine gut microbiota might not be evident until 4–6 days following dietary change (Fernandes et al., 2014; Van Den Berg et al., 2013). Ideally, mares maintained on one diet could have been compared to groups of mares on differing diets. This was not possible in the current study nor does it reflect how broodmares are generally managed on many stud farms. Transition between these diets was abrupt, and it was not known exactly when the faecal microbiota changed in relation to this.

## Conclusions

This is the first study to perform a detailed investigation of faecal microbiota and faecal metabolome of a group of mares during the periparturient period when they are at increased risk of colic. The study demonstrated that the majority of changes identified in the faecal microbiota and VOCs were mare-specific, and did not appear to be related to inherent physiological changes associated with foaling. The change in three VOCs post-parturition is interesting and may reflect a subtle change in the functional microbiome. Further studies are warranted to identify mares with altered metabolome in the periparturient period to determine whether there is a link to the risk of colic. VOCs may provide a cost-effective means of monitoring such mares with the ultimate aim of developing stable-side tests to identify and monitor mares at increased risk of colic.

## Supplemental Information

10.7717/peerj.6687/supp-1 Supplemental Information 1 Demographics of the mares included in the study.

10.7717/peerj.6687/supp-2 Supplemental Information 2 Relative abundance of different bacterial groups identified in the mare faecal microbiota. Relative abundance of different bacterial groups identified in the faecal microbiota of periparturient mares at different sampling occasions.

10.7717/peerj.6687/supp-3 Supplemental Information 3 A list of OTUs that varied significantly before and after foaling. A list of OTUs that were either upregulated or downregulated post-foaling.

10.7717/peerj.6687/supp-4 Supplemental Information 4 Pictorial description of the sampling strategyused in the study. Pictorial description of the sampling strategy used in the study. The mares were fed three different types of forages during the study period (grass and hay (F1), grass only (F2) and hay only (F3)).

10.7717/peerj.6687/supp-5 Supplemental Information 5 Cluster analysis of microbiome data. Agglomerative hierarchical cluster analysis performed on a Bray–Curtis dissimilarity matrix calculated from the microbiome data. The Figure shows that samples are clustered by individual mares rather than by the sampling time points. Each colour refers to an individual mare and the numbers from −3 to 7 refer to the time of sampling relative to foaling. A dash indicates that the sample was collected prior to foaling.

10.7717/peerj.6687/supp-6 Supplemental Information 6 OTUs significantly changed after foaling. A dot plot of significantly differentially abundant OTUs between samples collected before (T-3–T-1) and after (T1–T3) foaling. Only 81 significantly differentially abundant OTUs were identified. Samples collected before foaling is the reference category.

10.7717/peerj.6687/supp-7 Supplemental Information 7 Box plots of the number of VOCs. Boxplots comparing the mean number of VOCs between the (A) types of feed, (B) individual mares, and (C) sampling time relative to foaling. These differences were not statistically significant.

## Additional Information and Declarations

### Data Availability

The following information was supplied regarding data availability: The raw sequence data, Qiime script and the R codes used for data analyses are available at figshare: Salem, Shebl (2019): A longitudinal study of the faecal microbiome and metabolome of periparturient mares. figshare. Fileset. DOI 10.6084/m9.figshare.7267343.v1. Sequence data are available at the NIH Sequence Read Archive, accession number PRJNA523602.

## References

[ref-1] Aggio R, Villas-Boas SG, Ruggiero K (2011). Metab: an R package for high-throughput analysis of metabolomics data generated by GC-MS. Bioinformatics.

[ref-2] Benjamini Y, Hochberg Y (1995). Controlling the false discovery rate: a practical and powerful approach to multiple testing. Journal of the Royal Statistical Society: Series B (Methodological).

[ref-3] Blackmore TM, Dugdale A, Argo CMG, Curtis G, Pinloche E, Harris PA, Worgan HJ, Girdwood SE, Dougal K, Newbold CJ, McEwan NR (2013). Strong stability and host specific bacterial community in faeces of ponies. PLOS ONE.

[ref-4] Bray JR, Curtis JT (1957). An ordination of the upland forest communities of Southern Wisconsin. Ecological Monographs.

[ref-5] Cai L, Koziel JA, Davis J, Lo YC, Xin H (2006). Characterization of volatile organic compounds and odors by in-vivo sampling of beef cattle rumen gas, by solid-phase microextraction, and gas chromatography–mass spectrometry–olfactometry. Analytical and Bioanalytical Chemistry.

[ref-6] Caporaso JG, Bittinger K, Bushman FD, DeSantis TZ, Andersen GL, Knight R (2010a). PyNAST: a flexible tool for aligning sequences to a template alignment. Bioinformatics.

[ref-7] Caporaso JG, Kuczynski J, Stombaugh J, Bittinger K, Bushman FD, Costello EK, Fierer N, Pena AG, Goodrich JK, Gordon JI, Huttley GA, Kelley ST, Knights D, Koenig JE, Ley RE, Lozupone CA, McDonald D, Muegge BD, Pirrung M, Reeder J, Sevinsky JR, Turnbaugh PJ, Walters WA, Widmann J, Yatsunenko T, Zaneveld J, Knight R (2010b). QIIME allows analysis of high-throughput community sequencing data. Nature Methods.

[ref-8] Chao A (1984). Non-parametric estimation of the number of classes in a population. Scandinavian Journal of Statistics.

[ref-9] Cohen ND, Gibbs PG, Woods AM (1999). Dietary and other management factors associated with colic in horses. Journal of the American Veterinary Medical Association.

[ref-10] Cohen ND, Matejka PL, Honnas CM, Hooper RN (1995). Case-control study of the association between various management factors and development of colic in horses. Texas equine colic study group. Journal of the American Veterinary Medical Association.

[ref-11] Cohen ND, Peloso JG (1996). Risk factors for history of previous colic and for chronic, intermittent colic in a population of horses. Journal of the American Veterinary Medical Association.

[ref-12] Costa MC, Silva G, Ramos RV, Staempfli HR, Arroyo LG, Kim P, Weese JS (2015). Characterization and comparison of the bacterial microbiota in different gastrointestinal tract compartments in horses. Veterinary Journal.

[ref-13] Daly K, Proudman CJ, Duncan SH, Flint HJ, Dyer J, Shirazi-Beechey SP (2012). Alterations in microbiota and fermentation products in equine large intestine in response to dietary variation and intestinal disease. British Journal of Nutrition.

[ref-14] De Weerth C, Fuentes S, Puylaert P, De Vos WM (2013). Intestinal microbiota of infants with colic: development and specific signatures. Pediatrics.

[ref-15] DeSantis TZ, Hugenholtz P, Larsen N, Rojas M, Brodie EL, Keller K, Huber T, Dalevi D, Hu P, Andersen GL (2006). Greengenes, a chimera-checked 16S rRNA gene database and workbench compatible with ARB. Applied and Environmental Microbiology.

[ref-16] DiGiulio DB, Callahan BJ, McMurdie PJ, Costello EK, Lyell DJ, Robaczewska A, Sun CL, Goltsman DS, Wong RJ, Shaw G, Stevenson DK, Holmes SP, Relman DA (2015a). Temporal and spatial variation of the human microbiota during pregnancy. http://statweb.stanford.edu/~susan/papers/PNASRR.html.

[ref-17] DiGiulio DB, Callahan BJ, McMurdie PJ, Costello EK, Lyell DJ, Robaczewska A, Sun CL, Goltsman DS, Wong RJ, Shaw G, Stevenson DK, Holmes SP, Relman DA (2015b). Temporal and spatial variation of the human microbiota during pregnancy. Proceedings of the National Academy of Sciences of the United States of America.

[ref-18] Dougal K, Harris PA, Edwards A, Pachebat JA, Blackmore TM, Worgan HJ, Newbold CJ (2012). A comparison of the microbiome and the metabolome of different regions of the equine hindgut. FEMS Microbiology Ecology.

[ref-19] Driscoll N, Baia P, Fischer AT, Brauer T, Klohnen A (2008). Large colon resection and anastomosis in horses: 52 cases (1996–2006). Equine Veterinary Journal.

[ref-20] Edgar RC (2010). Search and clustering orders of magnitude faster than BLAST. Bioinformatics.

[ref-21] Edgar RC, Haas BJ, Clemente JC, Quince C, Knight R (2011). UCHIME improves sensitivity and speed of chimera detection. Bioinformatics.

[ref-22] Elli M, Colombo O, Tagliabue A (2010). A common core microbiota between obese individuals and their lean relatives? Evaluation of the predisposition to obesity on the basis of the fecal microflora profile. Medical Hypotheses.

[ref-23] Ellis CM, Lynch TM, Slone DE, Hughes FE, Clark CK (2008). Survival and complications after large colon resection and end-to-end anastomosis for strangulating large colon volvulus in seventy-three horses. Veterinary Surgery.

[ref-24] Fernandes KA, Kittelmann S, Rogers CW, Gee EK, Bolwell CF, Bermingham EN, Thomas DG (2014). Faecal microbiota of forage-fed horses in New Zealand and the population dynamics of microbial communities following dietary change. PLOS ONE.

[ref-25] Fischer S, Trefz P, Bergmann A, Steffens M, Ziller M, Miekisch W, Schubert JS, Köhler H, Reinhold P (2015). Physiological variability in volatile organic compounds (VOCs) in exhaled breath and released from faeces due to nutrition and somatic growth in a standardized caprine animal model. Journal of Breath Research.

[ref-26] French NP, Smith J, Edwards GB, Proudman CJ (2002). Equine surgical colic: risk factors for postoperative complications. Equine Veterinary Journal.

[ref-27] Garner CE, Smith S, De Lacy Costello B, White P, Spencer R, Probert CS, Ratcliffe NM (2007). Volatile organic compounds from feces and their potential for diagnosis of gastrointestinal disease. FASEB Journal: Official Publication of the Federation of American Societies for Experimental Biology.

[ref-28] Hackett ES, Embertson RM, Hopper SA, Woodie JB, Ruggles AJ (2014). Duration of disease influences survival to discharge of Thoroughbred mares with surgically treated large colon volvulus. Equine Veterinary Journal.

[ref-29] Harrison IW (1988). Equine large intestinal volvulus a review of 124 cases. Veterinary Surgery.

[ref-30] Hough R, Archer D, Probert C (2018). A comparison of sample preparation methods for extracting volatile organic compounds (VOCs) from equine faeces using HS-SPME. Metabolomics.

[ref-31] Hudson JM, Cohen ND, Gibbs PG, Thompson JA (2001). Feeding practices associated with colic in horses. Journal of the American Veterinary Medical Association.

[ref-32] Kaneene JB, Miller R, Ross WA, Gallagher K, Marteniuk J, Rook J (1997). Risk factors for colic in the Michigan (USA) equine population. Preventive Veterinary Medicine.

[ref-33] Leng J, Proudman C, Darby A, Blow F, Townsend N, Miller A, Swann J (2018). Exploration of the fecal microbiota and biomarker discovery in equine grass sickness. Journal of Proteome Research.

[ref-34] Lozupone CA, Hamady M, Kelley ST, Knight R (2007). Quantitative and qualitative beta diversity measures lead to different insights into factors that structure microbial communities. Applied and Environmental Microbiology.

[ref-35] Maechler M, Rousseeuw P, Struyf A, Hubert M, Hornik K (2016). https://cran.r-project.org/web/packages/cluster/index.html.

[ref-36] McMurdie PJ, Holmes S (2013). phyloseq: an R package for reproducible interactive analysis and graphics of microbiome census data. PLOS ONE.

[ref-37] Oksanen J, Blanchet FG, Kindt R, Legendre P, Michen PR, O’Hara RB, Simpson GL, Solymos P, Stevens MHH, Wagner H (2015). https://cran.r-project.org/web/packages/vegan/index.html.

[ref-38] Pinheiro J, Bates D, DebRoy S, Sarkar D, R Core Team (2015). https://cran.r-project.org/web/packages/nlme/index.html.

[ref-39] Price MN, Dehal PS, Arkin AP (2010). FastTree 2–approximately maximum-likelihood trees for large alignments. PLOS ONE.

[ref-40] Proudman CJ, Hunter JO, Darby AC, Escalona EE, Batty C, Turner C (2015). Characterisation of the faecal metabolome and microbiome of Thoroughbred racehorses. Equine Veterinary Journal.

[ref-41] R Core Team (2014). R: a language and environment for statistical computing.

[ref-42] Salem SE, Maddox TW, Berg A, Antczak P, Ketley JM, Williams NJ, Archer DC (2018). Variation in faecal microbiota in a group of horses managed at pasture over a 12-month period. Scientific Reports.

[ref-43] Schulz S, Dickschat JS (2007). Bacterial volatiles: the smell of small organisms. Natural Product Reports.

[ref-44] Shannon CE (1948). A mathematical theory of communication. Bell System Technical Journal.

[ref-45] Snyder JR, Pascoe JR, Olander HJ, Spier SJ, Meagher DM, Bleifer DR (1989). Strangulating volvulus of the ascending colon in horses. Journal of the American Veterinary Medical Association.

[ref-46] Suthers JM, Pinchbeck GL, Proudman CJ, Archer DC (2013). Risk factors for large colon volvulus in the UK. Equine Veterinary Journal.

[ref-47] Turnbaugh PJ, Hamady M, Yatsunenko T, Cantarel BL, Duncan A, Ley RE, Sogin ML, Jones WJ, Roe BA, Affourtit JP, Egholm M, Henrissat B, Heath AC, Knight R, Gordon JI (2009). A core gut microbiome in obese and lean twins. Nature.

[ref-48] Van Den Berg M, Hoskin SO, Rogers CW, Grinberg A (2013). Fecal pH and microbial populations in Thoroughbred horses during transition from pasture to concentrate feeding. Journal of Equine Veterinary Science.

[ref-49] Wang Q, Garrity GM, Tiedje JM, Cole JR (2007). Naive Bayesian classifier for rapid assignment of rRNA sequences into the new bacterial taxonomy. Applied and Environmental Microbiology.

[ref-50] Weese JS, Holcombe SJ, Embertson RM, Kurtz KA, Roessner HA, Jalali M, Wismer SE (2015). Changes in the faecal microbiota of mares precede the development of post partum colic. Equine Veterinary Journal.

[ref-51] Weiss SJ, Xu Z, Amir A, Peddada S, Bittinger K, Gonzalez A, Lozupone C, Zaneveld JR, Vazquez-Baeza Y, Birmingham A, Knight R (2015). Effects of library size variance, sparsity, and compositionality on the analysis of microbiome data. PeerJ PrePrints.

[ref-52] Wickham H (2009). ggplot2: elegant graphics for data analysis.

