# Supplementary material for: A longitudinal study of the faecal microbiome and metabolome of periparturient mares
Source: PeerJ. 2019 Apr 3;7:e6687. doi: 10.7717/peerj.6687 (PMC6451438; doi:10.7717/peerj.6687)
Supplement: Supplemental Information 1 [file peerj-07-6687-s001.docx]

**Table S1:** The demographics of the mares included in the study

| Mare | Breed | Age (years) | Height (hands) | Foaling due date | Date foaled | Days between foaling and T-3 | Days between foaling and T-1 | bred post foaling |
| --- | --- | --- | --- | --- | --- | --- | --- | --- |
| 1 | TB | 13 | 16 | 22/05/2014 | 25/05/2014 | 18 | 4 | Yes |
| 2 | TBX | 13 | 16.2 | 11/06/2014 | 15/06/2014 | 18 | 4 | No |
| 3 | TBX | 10 | 16.1 | 20/06/2014 | 04/06/2014 | 14 | 1 | Yes |
| 4 | WB | 7 | 16.1 | 22/06/2014 | 20/06/2014 | 16 | 2 | No |
| 5 | TBX | 12 | 16.1 | 2/07/2014 | 26/6/2014 | 16 | 1 | No |

TB = Thoroughbred, TBX = Thoroughbred cross, WB = Warmblood, T-3 = 1^st^ set of samples collected, T-1 = samples collected just before foaling
