# Supplementary material for: A longitudinal study of the faecal microbiome and metabolome of periparturient mares
Source: PeerJ. 2019 Apr 3;7:e6687. doi: 10.7717/peerj.6687 (PMC6451438; doi:10.7717/peerj.6687)
Supplement: Supplemental Information 2 — Relative abundance of different bacterial groups identified in the faecal microbiota of periparturient mares at different sampling occasions. [file peerj-07-6687-s002.docx]

**Table S2:**

1. Relative abundance (%) of different bacterial phyla identified in the faecal microbiota of periparturient mares at 11 time points

| **Phylum** | **T-3** | **T-2** | **T-1** | **T0** | **T1** | **T2** | **T3** | **T4** | **T5** | **T6** | **T7** | **Mean** |
| --- | --- | --- | --- | --- | --- | --- | --- | --- | --- | --- | --- | --- |
| Firmicutes | 43.126 | 40.922 | 36.687 | 42.318 | 37.520 | 36.078 | 38.221 | 37.963 | 42.534 | 39.035 | 40.866 | 39.570 |
| Bacteroidetes | 37.110 | 38.715 | 39.171 | 37.472 | 37.056 | 40.978 | 38.159 | 43.330 | 39.685 | 42.497 | 38.713 | 39.353 |
| Spirochaetes | 7.658 | 8.758 | 10.520 | 9.829 | 12.337 | 9.959 | 9.750 | 7.117 | 7.089 | 8.645 | 7.946 | 9.055 |
| Fibrobacteres | 5.515 | 5.138 | 7.185 | 4.536 | 5.874 | 5.826 | 6.704 | 4.783 | 3.399 | 3.195 | 4.818 | 5.179 |
| Verrucomicrobia | 2.337 | 1.629 | 2.486 | 1.913 | 2.399 | 2.885 | 2.560 | 2.308 | 2.649 | 2.017 | 3.089 | 2.388 |
| Proteobacteria | 2.045 | 2.703 | 2.162 | 1.971 | 2.356 | 1.962 | 2.027 | 1.852 | 1.642 | 2.083 | 1.645 | 2.041 |
| Tenericutes | 0.706 | 0.678 | 0.465 | 0.511 | 0.628 | 0.580 | 0.711 | 0.667 | 1.258 | 1.020 | 1.087 | 0.756 |
| TM7 | 0.283 | 0.366 | 0.263 | 0.273 | 0.273 | 0.262 | 0.328 | 0.293 | 0.304 | 0.270 | 0.254 | 0.288 |
| Cyanobacteria | 0.229 | 0.111 | 0.118 | 0.208 | 0.252 | 0.307 | 0.341 | 0.455 | 0.328 | 0.412 | 0.393 | 0.287 |
| Lentisphaerae | 0.193 | 0.110 | 0.282 | 0.282 | 0.397 | 0.130 | 0.319 | 0.417 | 0.281 | 0.230 | 0.383 | 0.275 |
| Unassigned | 0.367 | 0.360 | 0.205 | 0.222 | 0.211 | 0.222 | 0.296 | 0.249 | 0.218 | 0.174 | 0.268 | 0.254 |
| Synergistetes | 0.148 | 0.243 | 0.227 | 0.120 | 0.172 | 0.278 | 0.180 | 0.156 | 0.243 | 0.146 | 0.276 | 0.199 |
| Elusimicrobia | 0.077 | 0.065 | 0.027 | 0.037 | 0.255 | 0.145 | 0.150 | 0.197 | 0.115 | 0.113 | 0.078 | 0.114 |
| Actinobacteria | 0.135 | 0.085 | 0.096 | 0.190 | 0.113 | 0.128 | 0.099 | 0.112 | 0.116 | 0.078 | 0.098 | 0.114 |
| Armatimonadetes | 0.063 | 0.071 | 0.071 | 0.076 | 0.139 | 0.229 | 0.130 | 0.093 | 0.126 | 0.080 | 0.081 | 0.105 |
| WPS-2 | 0.004 | 0.043 | 0.030 | 0.034 | 0.017 | 0.027 | 0.012 | 0.004 | 0.005 | 0.001 | 0.002 | 0.016 |
| Planctomycetes | 0.005 | 0.002 | 0.004 | 0.007 | 0.001 | 0.005 | 0.012 | 0.005 | 0.008 | 0.002 | 0.002 | 0.005 |

1. Relative abundance (%) of different bacterial families identified in the faecal microbiota of periparturient mares at 11 time points

| **Family** | **T-3** | **T-2** | **T-1** | **T0** | **T1** | **T2** | **T3** | **T4** | **T5** | **T6** | **T7** | **Mean** |
| --- | --- | --- | --- | --- | --- | --- | --- | --- | --- | --- | --- | --- |
| Ruminococcaceae | 28.987 | 26.401 | 24.490 | 26.989 | 21.939 | 20.079 | 24.338 | 23.505 | 26.776 | 25.293 | 25.078 | 24.898 |
| Spirochaetaceae | 6.762 | 7.688 | 9.558 | 9.027 | 11.439 | 9.228 | 8.907 | 6.417 | 6.267 | 7.947 | 7.258 | 8.227 |
| Fibrobacteraceae | 5.515 | 5.138 | 7.185 | 4.536 | 5.874 | 5.826 | 6.704 | 4.783 | 3.399 | 3.195 | 4.818 | 5.179 |
| [Paraprevotellaceae] | 3.851 | 5.681 | 3.294 | 4.822 | 4.418 | 5.149 | 5.371 | 6.860 | 5.231 | 4.727 | 4.371 | 4.889 |
| Prevotellaceae | 3.317 | 3.205 | 2.732 | 4.000 | 3.079 | 3.896 | 4.785 | 4.136 | 2.903 | 3.532 | 3.178 | 3.524 |
| Veillonellaceae | 3.262 | 3.097 | 2.826 | 3.669 | 3.469 | 3.982 | 3.837 | 3.902 | 3.476 | 2.942 | 3.333 | 3.436 |
| Lachnospiraceae | 2.827 | 2.905 | 2.494 | 4.066 | 3.772 | 4.027 | 2.638 | 3.139 | 3.587 | 3.308 | 3.020 | 3.253 |
| Erysipelotrichaceae | 2.880 | 3.587 | 2.704 | 2.097 | 3.622 | 2.821 | 2.632 | 2.521 | 3.286 | 2.739 | 3.251 | 2.922 |
| RFP12 | 2.317 | 1.614 | 2.478 | 1.884 | 2.384 | 2.836 | 2.531 | 2.292 | 2.644 | 2.014 | 3.079 | 2.370 |
| BS11 | 2.006 | 2.210 | 2.374 | 1.262 | 1.079 | 1.298 | 1.690 | 0.972 | 2.689 | 3.828 | 4.893 | 2.209 |
| Unassigned | 0.901 | 0.893 | 0.943 | 0.861 | 0.883 | 0.944 | 0.845 | 0.968 | 0.941 | 0.974 | 0.845 | 0.909 |
| RF16 | 0.814 | 0.840 | 0.836 | 0.803 | 1.011 | 0.815 | 0.742 | 0.899 | 0.508 | 0.600 | 0.681 | 0.777 |
| Sphaerochaetaceae | 0.846 | 0.925 | 0.927 | 0.711 | 0.841 | 0.590 | 0.807 | 0.665 | 0.796 | 0.681 | 0.654 | 0.768 |
| Bacteroidaceae | 0.304 | 0.536 | 0.523 | 0.424 | 0.711 | 0.836 | 0.612 | 0.527 | 0.462 | 0.309 | 0.397 | 0.513 |
| [Mogibacteriaceae] | 0.423 | 0.438 | 0.322 | 0.386 | 0.459 | 0.573 | 0.477 | 0.546 | 0.563 | 0.453 | 0.453 | 0.463 |
| S24-7 | 0.476 | 0.543 | 0.351 | 0.410 | 0.410 | 0.412 | 0.235 | 0.273 | 0.314 | 0.267 | 0.453 | 0.377 |
| Clostridiaceae | 0.249 | 0.315 | 0.207 | 0.239 | 0.122 | 0.312 | 0.365 | 0.360 | 0.402 | 0.260 | 0.511 | 0.304 |
| Christensenellaceae | 0.289 | 0.291 | 0.260 | 0.242 | 0.355 | 0.237 | 0.269 | 0.341 | 0.367 | 0.233 | 0.295 | 0.289 |
| F16 | 0.283 | 0.366 | 0.263 | 0.273 | 0.273 | 0.262 | 0.328 | 0.293 | 0.304 | 0.270 | 0.254 | 0.288 |
| Marinilabiaceae | 0.143 | 0.304 | 0.237 | 0.353 | 0.182 | 0.214 | 0.184 | 0.214 | 0.129 | 0.220 | 0.156 | 0.212 |
| Victivallaceae | 0.079 | 0.056 | 0.180 | 0.152 | 0.282 | 0.051 | 0.183 | 0.194 | 0.145 | 0.103 | 0.155 | 0.144 |
| Porphyromonadaceae | 0.038 | 0.051 | 0.104 | 0.249 | 0.097 | 0.249 | 0.076 | 0.326 | 0.034 | 0.146 | 0.102 | 0.134 |
| R4-45B | 0.113 | 0.054 | 0.103 | 0.130 | 0.115 | 0.079 | 0.136 | 0.223 | 0.136 | 0.128 | 0.228 | 0.131 |
| Synergistaceae | 0.099 | 0.170 | 0.083 | 0.047 | 0.077 | 0.181 | 0.083 | 0.083 | 0.131 | 0.087 | 0.149 | 0.108 |
| Coriobacteriaceae | 0.131 | 0.081 | 0.096 | 0.148 | 0.112 | 0.122 | 0.098 | 0.109 | 0.100 | 0.071 | 0.091 | 0.105 |
| Mycoplasmataceae | 0.015 | 0.051 | 0.048 | 0.033 | 0.141 | 0.163 | 0.076 | 0.065 | 0.096 | 0.065 | 0.402 | 0.105 |
| Desulfovibrionaceae | 0.097 | 0.073 | 0.089 | 0.092 | 0.105 | 0.113 | 0.103 | 0.100 | 0.097 | 0.149 | 0.103 | 0.102 |
| Anaeroplasmataceae | 0.063 | 0.053 | 0.073 | 0.071 | 0.104 | 0.080 | 0.080 | 0.157 | 0.089 | 0.151 | 0.098 | 0.093 |
| Planococcaceae | 0.125 | 0.019 | 0.002 | 0.294 | 0.002 | 0.076 | 0.005 | 0.015 | 0.015 | 0.043 | 0.396 | 0.090 |
| Leuconostocaceae | 0.031 | 0.001 | 0.015 | 0.014 | 0.057 | 0.047 | 0.013 | 0.005 | 0.004 | 0.007 | 0.546 | 0.067 |
| Streptococcaceae | 0.000 | 0.002 | 0.001 | 0.011 | 0.302 | 0.117 | 0.089 | 0.059 | 0.005 | 0.000 | 0.007 | 0.054 |
| Alcaligenaceae | 0.032 | 0.040 | 0.056 | 0.058 | 0.085 | 0.041 | 0.052 | 0.037 | 0.046 | 0.032 | 0.058 | 0.049 |
| Eubacteriaceae | 0.043 | 0.028 | 0.044 | 0.051 | 0.025 | 0.061 | 0.045 | 0.028 | 0.058 | 0.040 | 0.046 | 0.043 |
| Campylobacteraceae | 0.073 | 0.060 | 0.040 | 0.040 | 0.009 | 0.045 | 0.076 | 0.025 | 0.024 | 0.032 | 0.024 | 0.041 |
| Enterobacteriaceae | 0.000 | 0.001 | 0.004 | 0.000 | 0.235 | 0.086 | 0.020 | 0.021 | 0.004 | 0.002 | 0.017 | 0.035 |
| Bacillaceae | 0.006 | 0.004 | 0.001 | 0.224 | 0.001 | 0.006 | 0.000 | 0.001 | 0.002 | 0.001 | 0.022 | 0.024 |
| Dethiosulfovibrionaceae | 0.008 | 0.015 | 0.037 | 0.041 | 0.031 | 0.017 | 0.026 | 0.015 | 0.011 | 0.009 | 0.047 | 0.023 |
| Dehalobacteriaceae | 0.018 | 0.037 | 0.022 | 0.015 | 0.013 | 0.032 | 0.018 | 0.008 | 0.028 | 0.015 | 0.022 | 0.021 |
| Rikenellaceae | 0.007 | 0.018 | 0.002 | 0.011 | 0.033 | 0.031 | 0.028 | 0.017 | 0.022 | 0.005 | 0.032 | 0.019 |
| Lactobacillaceae | 0.040 | 0.009 | 0.008 | 0.014 | 0.012 | 0.032 | 0.011 | 0.004 | 0.012 | 0.015 | 0.017 | 0.016 |
| Succinivibrionaceae | 0.011 | 0.020 | 0.004 | 0.033 | 0.026 | 0.020 | 0.013 | 0.011 | 0.014 | 0.008 | 0.012 | 0.016 |
| WCHB1-25 | 0.018 | 0.014 | 0.008 | 0.028 | 0.001 | 0.002 | 0.009 | 0.002 | 0.002 | 0.002 | 0.009 | 0.009 |
| Peptococcaceae | 0.017 | 0.009 | 0.002 | 0.024 | 0.007 | 0.005 | 0.007 | 0.002 | 0.000 | 0.005 | 0.005 | 0.008 |
| Moraxellaceae | 0.002 | 0.000 | 0.001 | 0.051 | 0.001 | 0.015 | 0.001 | 0.000 | 0.001 | 0.000 | 0.004 | 0.007 |
| p-2534-18B5 | 0.044 | 0.001 | 0.001 | 0.013 | 0.002 | 0.002 | 0.006 | 0.000 | 0.001 | 0.000 | 0.001 | 0.007 |
| [Weeksellaceae] | 0.000 | 0.001 | 0.001 | 0.043 | 0.004 | 0.001 | 0.001 | 0.000 | 0.000 | 0.000 | 0.005 | 0.005 |
| Oxalobacteraceae | 0.006 | 0.014 | 0.007 | 0.006 | 0.002 | 0.005 | 0.001 | 0.001 | 0.004 | 0.004 | 0.002 | 0.005 |
| Elusimicrobiaceae | 0.002 | 0.001 | 0.000 | 0.002 | 0.004 | 0.009 | 0.004 | 0.022 | 0.000 | 0.004 | 0.004 | 0.005 |
| Micrococcaceae | 0.004 | 0.000 | 0.000 | 0.041 | 0.000 | 0.002 | 0.000 | 0.000 | 0.000 | 0.000 | 0.002 | 0.005 |
| Cyclobacteriaceae | 0.002 | 0.000 | 0.004 | 0.001 | 0.001 | 0.000 | 0.007 | 0.000 | 0.000 | 0.000 | 0.001 | 0.002 |
| Microbacteriaceae | 0.000 | 0.001 | 0.000 | 0.000 | 0.000 | 0.000 | 0.000 | 0.000 | 0.008 | 0.001 | 0.004 | 0.001 |
| Nocardiaceae | 0.000 | 0.000 | 0.000 | 0.000 | 0.000 | 0.002 | 0.000 | 0.001 | 0.002 | 0.005 | 0.000 | 0.001 |
| Mycobacteriaceae | 0.000 | 0.000 | 0.000 | 0.000 | 0.001 | 0.000 | 0.001 | 0.001 | 0.005 | 0.001 | 0.000 | 0.001 |
| Corynebacteriaceae | 0.000 | 0.002 | 0.000 | 0.001 | 0.000 | 0.001 | 0.000 | 0.001 | 0.000 | 0.000 | 0.001 | 0.001 |
| Enterococcaceae | 0.000 | 0.000 | 0.000 | 0.002 | 0.000 | 0.000 | 0.001 | 0.000 | 0.001 | 0.000 | 0.001 | 0.001 |
