# Supplementary figures and images for: A longitudinal study of the faecal microbiome and metabolome of periparturient mares
Source: PeerJ. 2019 Apr 3;7:e6687. doi: 10.7717/peerj.6687 (PMC6451438; doi:10.7717/peerj.6687)

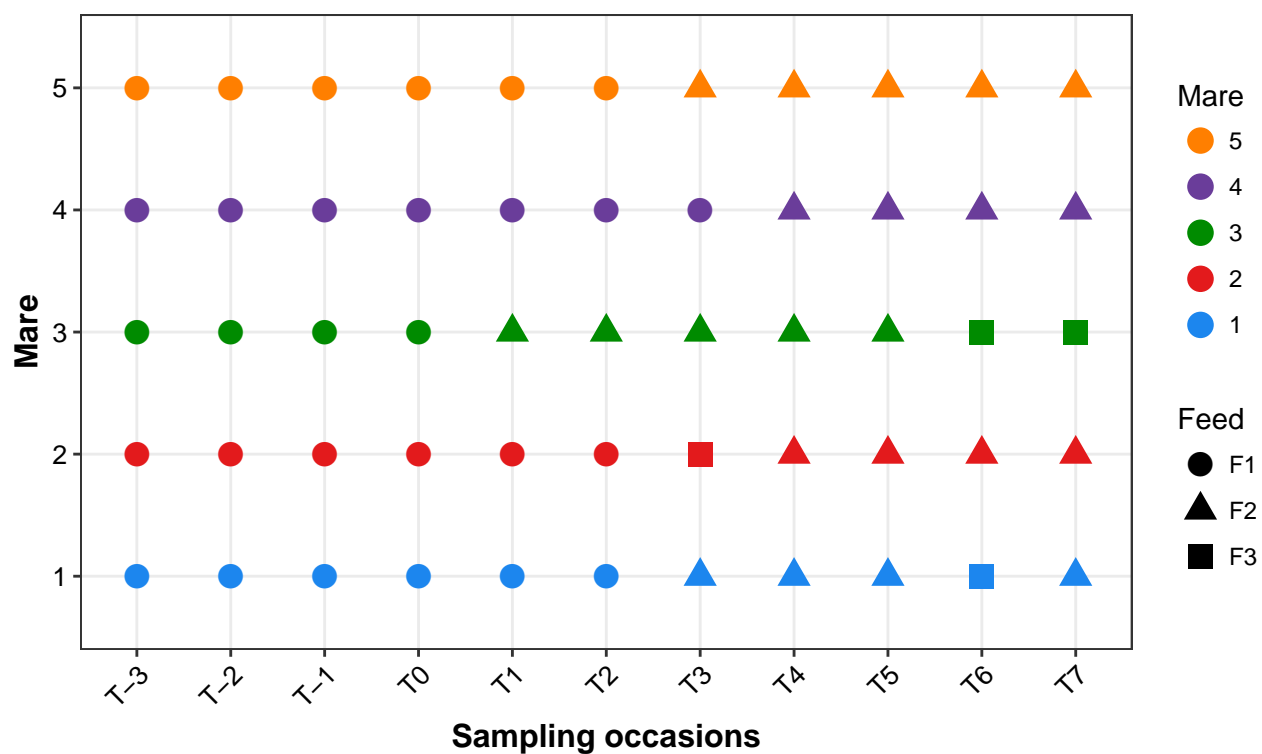

Supplement: Supplemental Information 4 — Pictorial description of the sampling strategy used in the study. The mares were fed three different types of forages during the study period (grass and hay (F1), grass only (F2) and hay only (F3)). [file peerj-07-6687-s004.pdf]

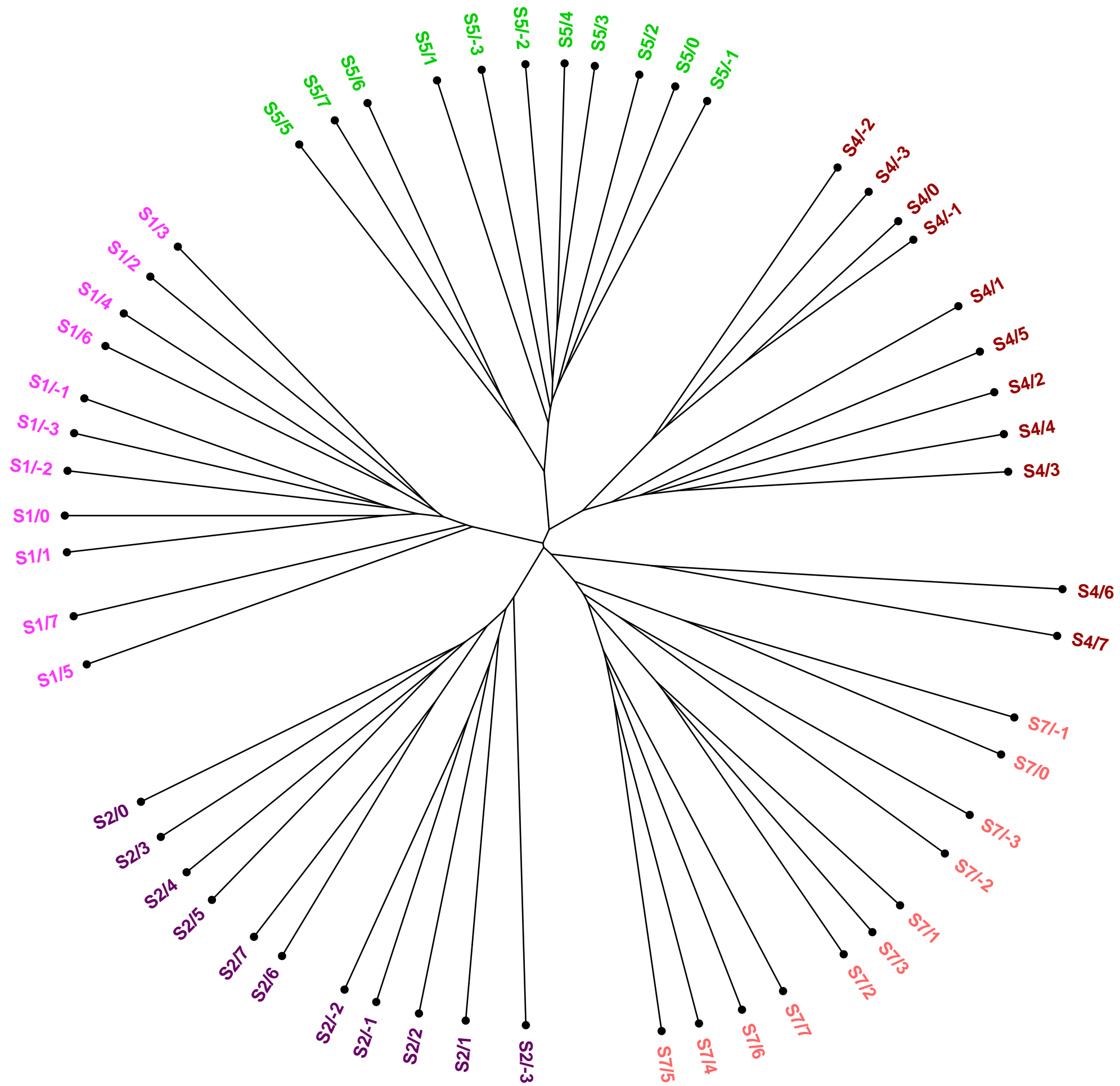

Supplement: Supplemental Information 5 — Agglomerative hierarchical cluster analysis performed on a Bray–Curtis dissimilarity matrix calculated from the microbiome data. The Figure shows that samples are clustered by individual mares rather than by the sampling time points. Each colour refers to an individual mare and the numbers from −3 to 7 refer to the time of sampling relative to foaling. A dash indicates that the sample was collected prior to foaling. [file peerj-07-6687-s005.pdf]

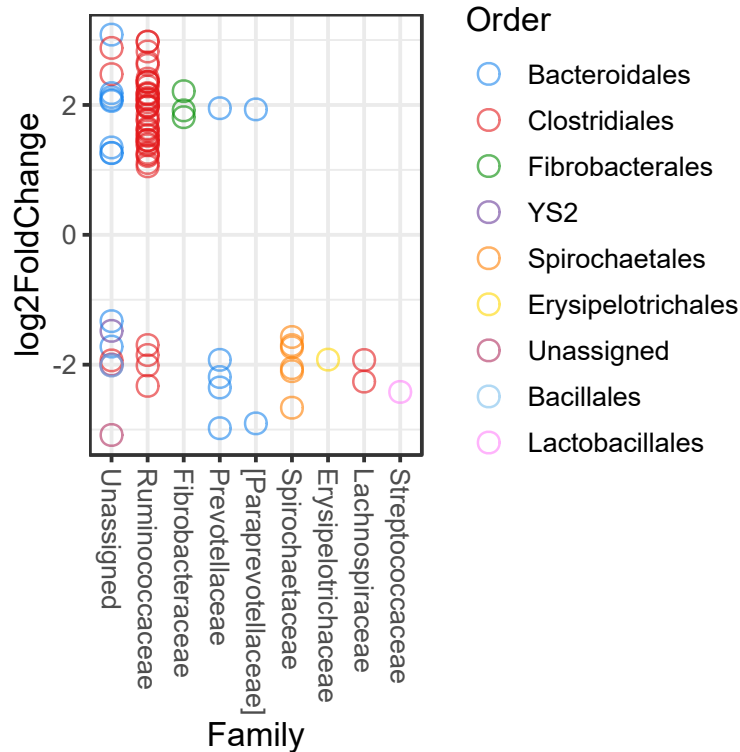

Supplement: Supplemental Information 6 — A dot plot of significantly differentially abundant OTUs between samples collected before (T-3–T-1) and after (T1–T3) foaling. Only 81 significantly differentially abundant OTUs were identified. Samples collected before foaling is the reference category. [file peerj-07-6687-s006.pdf]
