# Supplementary material for: A longitudinal study of the faecal microbiome and metabolome of periparturient mares
Source: PeerJ. 2019 Apr 3;7:e6687. doi: 10.7717/peerj.6687 (PMC6451438; doi:10.7717/peerj.6687)
Supplement: Supplemental Information 7 — Boxplots comparing the mean number of VOCs between the (A) types of feed, (B) individual mares, and (C) sampling time relative to foaling. These differences were not statistically significant. [file peerj-07-6687-s007.pdf]

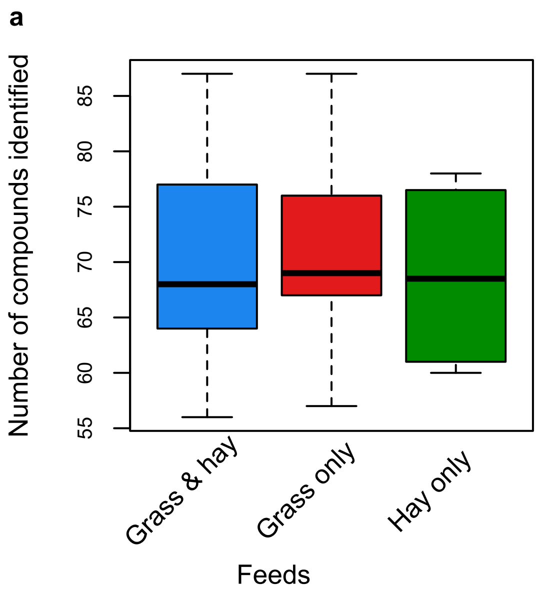

a
Number of compounds identified
55
60
65
70
75
80
85
Grass & hay
Grass only
Hay only
Feeds

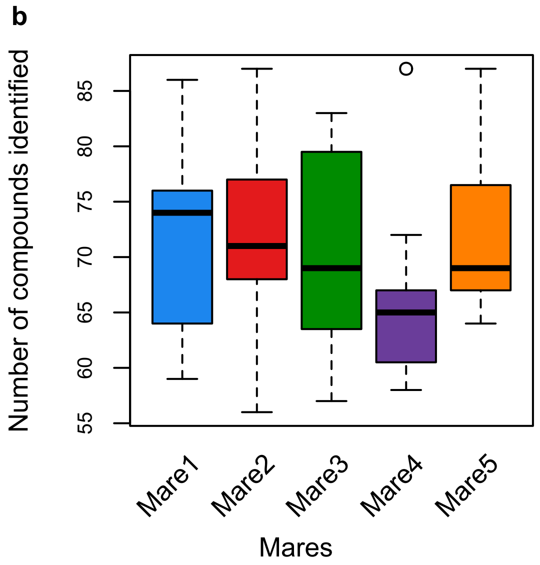
b
Number of compounds identified
55
60
65
70
75
80
85
Mare1
Mare2
Mare3
Mare4
Mare5
Mares

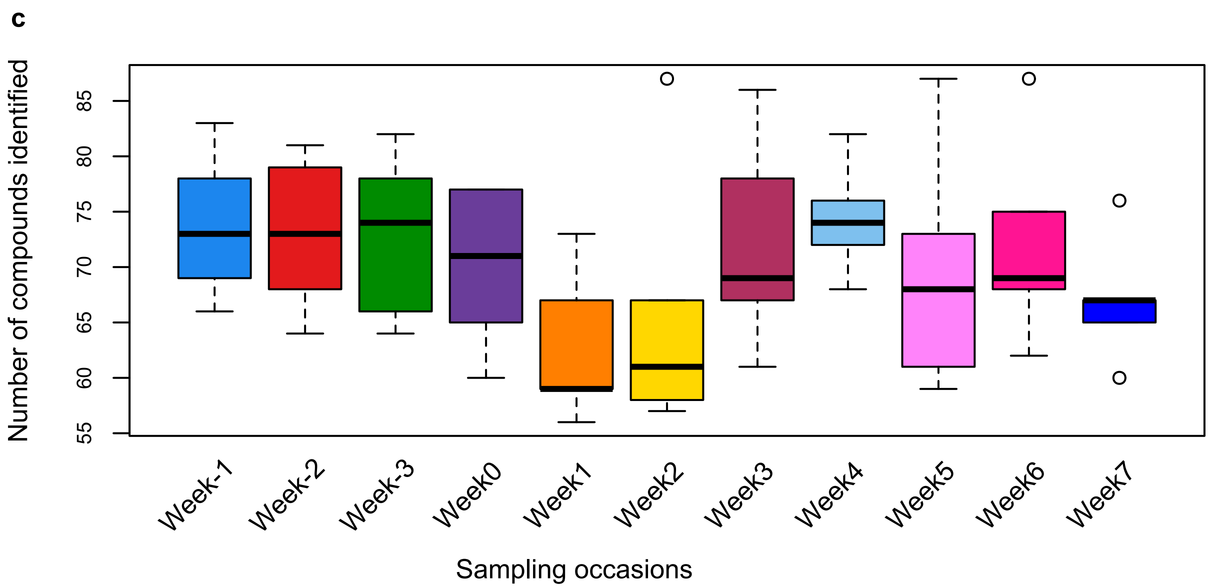
c
Number of compounds identified
55
60
65
70
75
80
85
Week-1
Week-2
Week-3
Week0
Week1
Week2
Week3
Week4
Week5
Week6
Week7
Sampling occasions
